# “Nowhere to go” qualitative exploration of social workers' perspectives on violence against women in Jordan during the COVID-19 pandemic

**DOI:** 10.3389/fgwh.2026.1899338

**Published:** 2026-09-24

**Authors:** Hana Taha, Asma Basha, Mohammed Nawaiseh, Bashar Tarabsheh, Zaid Akhras, Awn Al-Shehab, Zaid Abu-Ameereh, Abdulla Al-hawamdeh, Vanja Berggren

**Affiliations:** 1 Department of Family and Community Medicine, School of Medicine, The University of Jordan, Amman, Jordan; 2 Department of Neurobiology, Care Sciences and Society, Karolinska Institutet, Stockholm, Sweden; 3 Department of Obstetrics and Gynecology, School of Medicine, The University of Jordan, Amman, Jordan; 4 Department of Ophthalmology, Jordanian Royal Medical Services, Amman, Jordan; 5 Department of Anesthesia, Islamic Hospital, Amman, Jordan; 6 Department of Internal Medicine, Suleiman Al-Habib Medical Group, Riyadh, Saudi Arabia; 7 Department of Health Care Sciences, Marie Cederskiöld University, Stocholm, Sweden

**Keywords:** COVID-19, domestic violence, gender based violence, Jordan, social workers

## Abstract

**Background:**

The COVID-19 pandemic significantly changed global socio-economic aspects of many humans' lives, leading to an increased risk of domestic violence (DV). This study aims to explore the impact of the COVID-19 pandemic on the dynamics of DV in Jordan by exploring the social workers' perceptions of its underlying causes and how the unique socio-economic and psychological strains of the pandemic affected DV within the Jordanian context.

**Methods:**

Qualitative study conducted from February to July 2022 in Amman, Jordan. The data were collected through individual in-depth interviews with 20 social workers who work in different Family and Juvenile Protection centers throughout Amman city, to explore their perceptions about the status of violence against women during the coronavirus pandemic. The data was analyzed using latent content analysis.

**Findings:**

The researchers were able to recognize two themes from the narratives; The first theme was: COVID-19 fear and restrictions, aggravated the barriers and impacted reaching out and access to social services. This theme was built on two categories: Prevailing socio-cultural hushing especially among less privileged women; the victim's ambivalence between the risk of exposure to the virus versus her fear from the perpetrator. The second theme was: COVID-19 pandemic and restrictions generated tremendous stress for victims, perpetrators, and social workers. This theme was constructed on four categories: the increase in the incidence of domestic abuse during the pandemic; the increase of socio-economic hardships; the increase in alcohol and drug abuse by victims and their abusers; and the social workers' concern about the accessibility to ask for help during the quarantine.

**Conclusion:**

There has been a noticeable increase in DV in Jordan during COVID-19 crisis attributed to many factors such as spending a lot of time with the abusers and physical barriers for accessing family protection departments. Empowering women and providing psychosocial support to both victims and perpetrators can prevent and decrease the hazardous impact of DV. Digital reporting tools and standardized tele-counseling protocols for social workers should be implemented to overcome the possible physical and socio-economic barriers of reporting.

## Introduction

From December 2019, when COVID-19 has been identified in Wuhan, to august 2021 more than 200 million COVID-19 cases have been recorded around the world, affecting almost every country in the world (1, 2). Countries around the globe started taking actions, such as encouraging individuals to adopt “social distancing”, mandating school and business closures, and imposing travel restrictions that may have reduced the transmission of the infectious disease (3, 4); unfortunately, these actions were also associated with increased incidence of domestic violence (DV), especially against women (5). Many DV victims may have faced the “worst case” scenario – they found themselves trapped in the home with a violent perpetrator during a time of severely limited contact with the outside world (5–7). Lockdown decreased the cases of COVID-19 but during the lockdown, many people suffered from a decrease in their income, stress, depression, and fear of getting infected (5).

DV is defined by the United Nation (UN) as “a pattern of behavior in any relationship that is used to gain or maintain power and control over an intimate partner” (8). DV can happen to anyone regardless of their gender, age, race, educational or economic level, and it ranges from physical harm to emotional and psychosocial manipulations (8). Generally, DV abusers tend to isolate their victims as an act of control. The pandemic situation provided a perfect environment for them. Scenarios similar to the COVID-19; in situations of rapidly increasing stress, sudden shifts in daily routines, closing of schools and community resources, and the rapid decrease in available resources as in natural disasters that showed DV reports increased (9) along with the increased in reported alcohol abuse, family stress, and aggression. Additionally, studies that indicate the increased rates of DV reported after a natural disaster (9) often extend for several months after the occurrence of the catastrophic event (7). Reports of increasing rates of DV surfaced around the world during the COVID-19 pandemic lockdowns, with rates rising between 30%–50% in certain countries (5–8, 10–13). In Jordan, there was an increase in DV incidents during the outbreak by 40% and more compared to the usual conditions. Also, the predictors of violence against women during COVID-19 were found to be unemployed and married (14).

This study will use the General Strain Theory (GST) to connect the study's purpose with its conceptual basis. Strains, such as income loss or forced quarantine, are associated with negative emotions like anger and frustration. In the absence of positive coping mechanisms, people may resort to criminal coping, including DV or substance abuse, as a response to these strains (15, 16).

While the acute restrictions of the COVID-19 pandemic have ended in, the crisis remains an important case study for understanding the resilience of social protection systems. This research retrospectively explores the pandemic as a stress test that amplified pre-existing socio-cultural and economic strains in Jordan.

There is a lack of information regarding DV against women during the COVID-19 era (6) and the majority of DV studies in Jordan are cross-sectional in nature and relied on measuring violence using an online self-administered survey (14, 17). These methods often capture manifest data without the broader systemic context. This study utilizes the social workers' perspectives to synthesize individual narratives into a broader systemic pattern, that victims may not be able to report. Moreover, their experience allowed them to interpret social and cultural nuances, evaluate the barriers that emerged during the pandemic, and bridge the gap between individual victim experiences and institutional responses. Thus, their views provide a more holistic understanding of the factors associated with increased DV during the pandemic that standardized surveys may overlook. This study aimed to explore social workers' perceptions about DV and its underlying causes during the COVID-19 pandemic in Jordan. To achieve this, the research explored how the unique socio-economic and psychological strains of the pandemic affected the dynamics of DV within the Jordanian context.

## Materials and methods

### Study setting

Jordan is a upper middle-income country with a population of 11 million (49% females) (18). Eight out of ten (83%) live in cities and the rest in rural areas and deserts. In 2021, the average Gross Domestic Product (GDP) per capita is 3,781.43 US $ (19). This study was conducted in one governorate; Amman which constitutes 60% of the total population and demonstrates the Jordanian's society socio-culture texture. Amman has a total population of 6.4 million (18, 20). There are clear socio-economic disparities between Amman's western and eastern parts. West Amman is the affluent side of the city, while East Amman is the underprivileged side of the city. People in the respective parts of the city have different lifestyles, experiences, beliefs, and perceptions (21). In 1997, the Public Security Directorate in Amman initiated the idea of the Family and Juvenile Protection Division (FJPD) to deal with DV and sexual abuse cases. Since then the FJPD has operated as multidisciplinary unit and spread to cover most of Jordan's governorates (22). The division works for the well-being of its clients through a network of facilities that are staffed with well trained personnel to meet the criteria of a friendly atmosphere for the victims (23). The COVID-19 pandemic catalyzed a strategic evolution from facility-based care to a decentralized, technology-reliant model, where the reporting intake could be done via the national 911 emergency system and social media platforms.

Interviewing victims is a human rights process that is conducted in a comfortable women-friendly facilities, not in formal settings to suit the women's psychosocial needs. Female police officers and female social workers investigate and interview female clients. Whereas Male offenders are investigated by male officers and male social workers (24). Before the Covid-19 pandemic, Jordan's Population and Family Health Survey found that 19% of women in reproductive age (15–49 years) had experienced sexual violence by an intimate partner at least once in a lifetime and 21% had experienced physical violence (25). Based on a fast assessment by the UN women in the beginning of the pandemic, 62% of the women felt an increased risk of physical or psychological violence due to stresses caused by Covid-19 (26). Table 1 shows the Findings from the UN women national survey in Jordan in 2021 that targeted women aged 18 and older who had access to a phone (Nearly 68% of the total population of women aged 18 years and older in Jordan (27).

**Table 1:** Findings of the UN women 2021 survey, that shows the effect of COVID-19 on violence against women.

Indicator/Measure		Percentage (%)	
Women who experienced a form of violence or know another woman who experienced violence	66%	Verbal abuse	42%
		Physical abuse	46%
		Sexual abuse	33%
Women who felt unsafe in their homes.	13%	Women who said this has gotten worse during the COVID-19 pandemic.	21%
Women who live in households with conflict among adults at least weekly.	38%	Women who said that conflict between adults has become more frequent during the COVID-19 pandemic.	39%
Women who said that the experience of verbal or physical abuse at the hands of a partner is common for women in their community	40%	Women who said this has gotten worse during the COVID-19 pandemic.	74%
Women who thought that physical harm, abuse and harassment are a problem for women in their community.	41%	Women who thought that physical harm, abuse, and harassment has gotten worse since the onset of the COVID-19 pandemic.	11%

Data adapted from UN Women (2021), “Measuring the Shadow Pandemic: Violence Against Women During COVID-19 Country Report: Jordan”. Available at: https://data.unwomen.org/publications/vaw-rga.

### Study design

To explore the perceptions of social workers about the effect of COVID-19 on DV in Jordan, if there was an increase in DV and what are the main causes for this increase, the researchers used qualitative methods to achieve that purpose. The research team developed an Arabic interview guide with open ended questions based on the literature (28–32). The guide was adjusted based on two pilot in-depth interviews with social workers from the FJPD centers in Amman. After that, the research team conducted 18 in-depth interviews until reaching data redundancy and saturation. The narratives of the 20 in-depth interviews including the pilot ones were transcribed in Arabic and included in the analysis. Data saturation was determined through a concurrent analysis process, where the research team reviewed transcripts immediately following each interview. Saturation was reached when redundancy of data occurred and no new codes, categories or themes emerged (33).

### Study population

To ensure the versatility of information, purposive sampling was used to recruit 20 participants (9 females and 11 males) (34). The sampling strategy deliberately included social workers who worked in the field and managers of FJPD centers to gather in-depth insights and achieve a comprehensive, multi-level organizational perspective. Social workers provided micro-level insights into psychological and behavioral dynamics of victims and perpetrators, while center managers supplied macro-level organizational perspectives on administrative obstacles, institutional policy adaptations, and workload changes.

Fifteen had a bachelor's degree and were specialized in psychosocial counseling (8 females aged between 27 and 33 and 7 males aged 30–51 years). The researchers also interviewed 5 managers in the different FJPD centers (One female aged 35 and 4 males aged 46–55). Social workers and center managers were selected as key informants rather than recruiting victims directly due to strict institutional protocols, ethical constraints, and safety risks associated with interviewing active DV victims during a public health crisis. The inclusion of both male and female professionals allowed for a balanced analytical perspective. The sample characteristics are described in Table 2:

**Table 2:** The sociodemographic characteristics of the key informants.

Key informants’ characteristics				
*Sex*	*Female*		*Male*	
	9		11	
*Age*	27–29	*N* = 3	30–35	*N* = 2
	30–35	*N* = 6	36–45	*N* = 2
			46–51	*N* = 5
			52–55	*N* = 2
*Field social workers*	8		7	
Managers of FJPD centres	1		4	
*Education*				
BSc	8		7	
Master	1		4	
*Specialization*				
Sociology (trained in psychosocial counselling)	8		7	
Applied Psychology	1		2	
Public management			2	

### Data collection

Data collection was conducted retrospectively between February and July 2022. This timeline was selected to capture frontline social workers' professional experiences in close temporal proximity to the lifting of major COVID-19 restrictions in Jordan (late September 2021) (35), thereby minimizing recall bias.

The data was collected from 4 FJPD centers in East and West Amman. The interviews took place in quiet rooms, most of which were the same as the victims were interviewed usually. Open-ended questions were used to encourage spontaneous dialogue with the social worker. All the interviews were done in Arabic. Each one lasted 25–30 min. Two researchers attended each interview, the first conducted the interview and the second took notes. Due to some security and confidentiality matters, only 4 of the interviews were audio recorded. The remaining interviews were written carefully in Arabic in an effort to include every word from the workers. All the data was transcribed in Arabic. To ensure maximum data accuracy and minimize loss of details, a standardized dual-investigator protocol was implemented: one researcher conducted the interview while the second researcher took detailed, near-verbatim field notes. Immediately following each interview, both researchers collaborated to review and finalize the Arabic transcripts to preserve exact expressions, context, and non-verbal nuances.

### Data analysis

Data analysis was conducted by bilingual Jordanian researchers fluent in English and native in Arabic, ensuring deep familiarity with local idioms and cultural context. The data were subjected to qualitative latent content analysis. The researchers condensed the Arabic text into meaning units followed by double coding in English by two researchers separately. An inductive (data-driven) approach was used to assign initial conceptual labels (codes) to the condensed meaning units. To ensure translation validity, a third researcher performed a back-translation check on a subset of the codes (approximately 20%) to ensure the original meanings were preserved. Any discrepancies were resolved by discussion until a consensus was reached. This was followed by final categorization and themes extraction.

Qualitative content analysis is an autonomous technique and can be used at varying levels of abstraction and interpretation. It carries the common methodological challenge of presenting the descriptions of manifest content and the latent interpretations. Increased abstraction and interpretation can jeopardize the credibility and trustworthiness of the analysis (36). Thus, the analysis of the key informants' data in this research retained the logic of how the categories and themes are abstracted, interpreted, and linked to the study aim and to each other. The researchers used a hybrid thematic approach, combining inductive coding (derived directly from the narratives) with a deductive framework informed by GST. This ensured that a theoretical framework informed the interpretation process.

During the inductive phase, primary coding was conducted by reviewing raw transcripts to extract initial codes directly from the participants' language and narrative context. This ensured that unique, emergent phenomena specific to the pandemic lockdown were captured without being constrained by preconceived theoretical frameworks. In the deductive phase, the codes and new themes were systematically organized onto a multi-tiered institutional framework. This hybrid approach preserved the authentic voices of social workers while placing their insights within a structured, system-level analytical framework.

### Ethical consideration

This research was ethically approved by The Hashemite University Institutional Review Board (IRB) committee. The confidentiality and autonomy of the participants were ensured. All the participants were given an informed consent paper in which they read it out loud and agreed to participate by signing it; The informed consent explained the purpose of the study, the voluntary nature of their participation, and their right to access findings.

Confidentiality was maintained by not collecting any identifying personal information, immediate pseudonymization using broad descriptive codes (e.g., “Female social worker”, “Male manager”), conducting interviews in private FJPD rooms, keeping handwritten notes in locked cabinets, and storing digital data on local encrypted drives.

## Findings

Two themes emerged during the data analysis, and they were constructed on the below categories as listed in Table 3.

**Table 3:** Themes and categories of social workers’ perceptions about domestic violence against women during the COVID-19 pandemic.

Themes	Categories
1. COVID-19 fear and restrictions, aggravated the barriers and impacted reaching out and access to social services	*A. Prevailing Socio-cultural hushing especially among less privileged women* *B. The victim's ambivalence between the risk of exposure to the virus versus her fear from the perpetrator.*
*2.* The COVID-19 pandemic and restrictions generated tremendous stress for victims, perpetrators, and social workers.	*A. The increase in the incidence of domestic abuse during the pandemic.* *B. The increase of socio-economic hardships* *C. The increase in alcohol and drug abuse by victims and their abusers* *D. The social workers’ concern about the accessibility to ask for help during the quarantine.*

### Theme 1: COVID-19 fear and restrictions, aggravated the barriers and impacted reaching out and access to social services

The first theme emerged from the participants narratives about the barriers that stopped women from seeking help and it was constructed on two categories: Prevailing Socio-cultural hushing especially among less privileged women and the victim's ambivalence between the risk of exposure to the virus versus her fear from the perpetrator.

#### Category A/1: prevailing socio-cultural hushing especially among less privileged women

In the narratives, the respondents said that in the Arab societies, women are frequently positioned as the more vulnerable party in marital conflicts, especially when the husband serves as the primary breadwinner who provides for her and the children. When a woman does not get the support from her family, this force her to stay with her abusive husband. She tolerates the harm and remains silent because the family and the culture of society do not provide her with support. This shows the systemic entrapment of victims, caused by the pandemic mandates that stripped women of viable alternatives for safety.

When we asked women, the answer was “Where do I go if I go out of the house? into the street! There is no other place that will accept me and my kids, which obliges me to stay”. Female social worker.

“Some women are trapped because they can't leave their kids, the husband hits her several times and when she escapes to her family home; they tell her, you can stay here without his kids” Male social worker.

The social workers said that DV undermines the health, dignity, security, and autonomy of its victims, but it goes unreported due to the stigma and shame that victims' experience. Most of women who seek help immediately look to close family and friends, due to the stigmatized perception of the situations they undergo. They reach out to FJPD centers when they can't get their family support to stop the abuser. The social workers indicated that the social and economic context can be quite restraining, where women are expected to keep her family together and mend the family ties. In many cases their care could be extended to include the elderly and those with disabilities in the family home. The data displayed that in the Jordanian context men rarely offer assistance in domestic work (37). With the COVID-19 restrictions and school closures, the drain on stay-at home mothers, and mothers working remotely from home has increased exponentially, this inevitably led to emotional and physical exhaustion.

“Women are the anchor for their children's physical, mental, and emotional wellbeing”. Female social worker.

“Even if a woman suffers from DV, she hides that to protect her family”. Male social worker.

“In our country there is a pressure on women to sacrifice her own wellbeing and keep the violence behind closed doors”. Female social worker.

“Divorced women are labeled by the society, so she tolerates the abusive husband to protect herself from disgrace” Female manager of a family protection center

This illustrates a culturally enforced mandate of self-sacrifice, where women act as the primary family “anchors” by suppressing their own wellbeing to preserve domestic stability, social reputation, and children's development.

#### Category B/1: the victim's ambivalence between the risk of exposure to the virus versus her fear from the perpetrator

The general closure has put the victims in an unenviable position, where they had one of two options, either to stay with the abuser or to go out and contract the Corona virus and to be exposed to disease and death, said one of the social workers.

“Victims came to us despite of their fear from the virus, but they had to escape from a greater danger, which is violence inside their home”. Male manager of a family protection center.

One of the abused women told the social workers:

“Death from the virus is more merciful than death from beating”. Male social worker.

“Women wanted to escape from the violence, but they were scared from the virus, and this kept them home with the abuser”. Male social worker.

“Being unsafe at home with a perpetrator was a familiar danger for these women but the virus was frightening because it was unpredictable”. Female social worker.

Together, these narratives demonstrate a highly fluid “hierarchy of danger”. Victims attempted balancing the acute threat of DV against the broader, unpredictable public health crisis of the pandemic. This balance dictated their physical ability to break quarantine to seek safety, which resulted in their entrapment within the home with the abuser.

Many abused women faced difficulties in reaching family protection centers due to lockdown during the pandemic which made leaving the house very difficult. This forced them to search for other means to communicate with protection centers through social media and phones or stay at home and live with the abuser while being exposed to violence continuously. The biggest threat to women's lives was not COVID-19, but the interruption of health services and women's fear of seeking protection from DV, where they could get infected.

During the COVID-19 pandemic outbreak, there was increased use of technology and employees were working remotely from home. However, general access to computers and mobile phones was restricted to women who suffered from violence specially in less privileged settings. During the outbreak, women's access to information and their ability to seek services were severely restricted, especially as the bulk of communication is conducted through online platforms and cell phone messaging. Poor women did not have access to internet nor to a mobile phone, which may have affected their ability to access comprehensive information about the crisis in terms of prevention, response and seeking help.

### Theme 2: the COVID-19 pandemic and restrictions generated tremendous stress for victims, perpetrators, and social workers

The second theme emerged from the interviewees' views about the increase in the incidence of domestic abuse during the pandemic was the increase of socio-economic hardships; the increase in alcohol and drug abuse by victims and their abusers; and the social workers' concern about the accessibility to ask for help during the quarantine.

#### Category A/2: the increase in the incidence of domestic abuse during the pandemic

The narratives revealed that there was an increase in the number of cases of violence during COVID-19. Females were the gender most exposed to violence who resorted to FJPD Centers.

“We noticed a significant increase in calls and messages to report cases of violence during the pandemic”. Manager of a family protection center.

“In Jordan and Arab societies in general, females are the weakest group in society and males always want to impose their opinions and decisions on them by force without the right to object”. Female social worker.

The reported increase in abuse cases during the pandemic may be attributed to the deeply embedded patriarchal power dynamic that uses force to maintain male dominance in response to external strains. Younger women in their early twenties are more vulnerable to violence than older women as mentioned by the social workers. Younger women were more targeted by the perpetrators because of power imbalance in the relationship where the male dominates and control the victim who cannot escape or make rational decisions.

“Most of the abused women are young”. Female social worker

“Being young makes those women less capable of escaping or reporting domestic violence”. Male manager of a family protection center

The participants said the main reason for the increase in DV during COVID19 is the imposition of curfews, which led to the family spending more time together and removing the protective buffer of physical distance, and this in itself leads to conflicts between family members and turning constant forced proximity into a direct catalyst for escalated DV.

“The more time people spend together, the greater the likelihood of violence”. Male social worker.

#### Category B/2: the increase of socio-economic hardships

This is attributed to the poor economic conditions during the pandemic, which led to the loss of jobs, in addition to the couples being forced to stay at home together for long periods due to the lockdown, and this generated stress. Husbands suffered because they were unable to secure the daily needs of their homes, with their inability to leave the house to search for work. For female survivors who have lost their jobs or source of income, attempting to leave an abusive partner became even more challenging during the COVID-19 pandemic due to financial dependence.

“The deterioration of the economic conditions could be behind the increase in the cases of violence”. Male Manager of a family protection center.

“More women experienced poverty during the COVID-19 pandemic, which exacerbated gender biases and domestic violence”. Female social worker.

“Some men used their ability to hurt their partners as a way to get some feeling of control”. Female social worker.

“The decrease in resources caused conflict between partners regarding the best way to spend the little amount of money left”. Male social worker.

This highlights that the economic decline and rising poverty during the pandemic acted as major stressors that worsened gender inequality, acted as primary source of household conflict, and fueled an increase in DV.

#### Category C/2: the increase in alcohol and drug abuse by victims and their abusers

To deal with stress and the constant pressure of uncertainty and fear, people would usually reach out for support from their friends and family members something that the quarantine made almost impossible for them and without that emotional support, many were left alone with the stress and the pressure created by the pandemic. The respondents said that there was an increase in the abuse of alcohol and drugs, whether by the abuser or the victim despite the lockdown because they had enough stock before the pandemic started.

“They had to maintain social distancing to combat the spread of COVID-19. The stress from fear and social isolation led to increase in drinking and substance abuse”. Male social worker.

“Stress and alcohol abuse led to increased domestic violence”. Female social worker.

Stress and fear from the virus triggered an increase in alcohol and substance abuse and acted as maladaptive coping mechanisms. This lowered behavioral inhibitions and increased DV.

#### Category D/2: social workers' concern about the accessibility to ask for help during the quarantine

The pandemic impacted the social workers' ability to address the increasing needs of DV victims in the context of the pandemic. They couldn't provide their counseling services as usual and had to adapt with the pandemic constraints. The social workers talked about battling to manage their workload during the crisis and their feeling of burnout.

“I was afraid to get COVID-19 but I also felt responsible to reach out to victims”. Female social worker.

“I had a moral commitment, but I was exhausted and needed help myself”. Female social worker.

“It was difficult to access protection centers due to the lockdown”. Male social worker.

This prompted the victims to use other methods to seek protection through the phone and social media. Those victims who managed to get access to a mobile phone also were able to seek help, as one of the social workers said:

“Most of the cases who reported violence were through the mobile phone, the public security emergency number 911, and Facebook”. Female social

The lockdown blocked the physical ordinary safety nets. This forced the burned-out social workers and desperate victims to utilize the underdeveloped digital protection services for reporting and responding to abuse cases.

To visually summarize the complex interplay of factors described by the social workers, the researchers developed a conceptual framework (Figure 1). This figure shows how the pandemic related strains and sociocultural mediators were associated with the observed increase in DV and related maladaptive coping outcomes.

**Figure 1:**
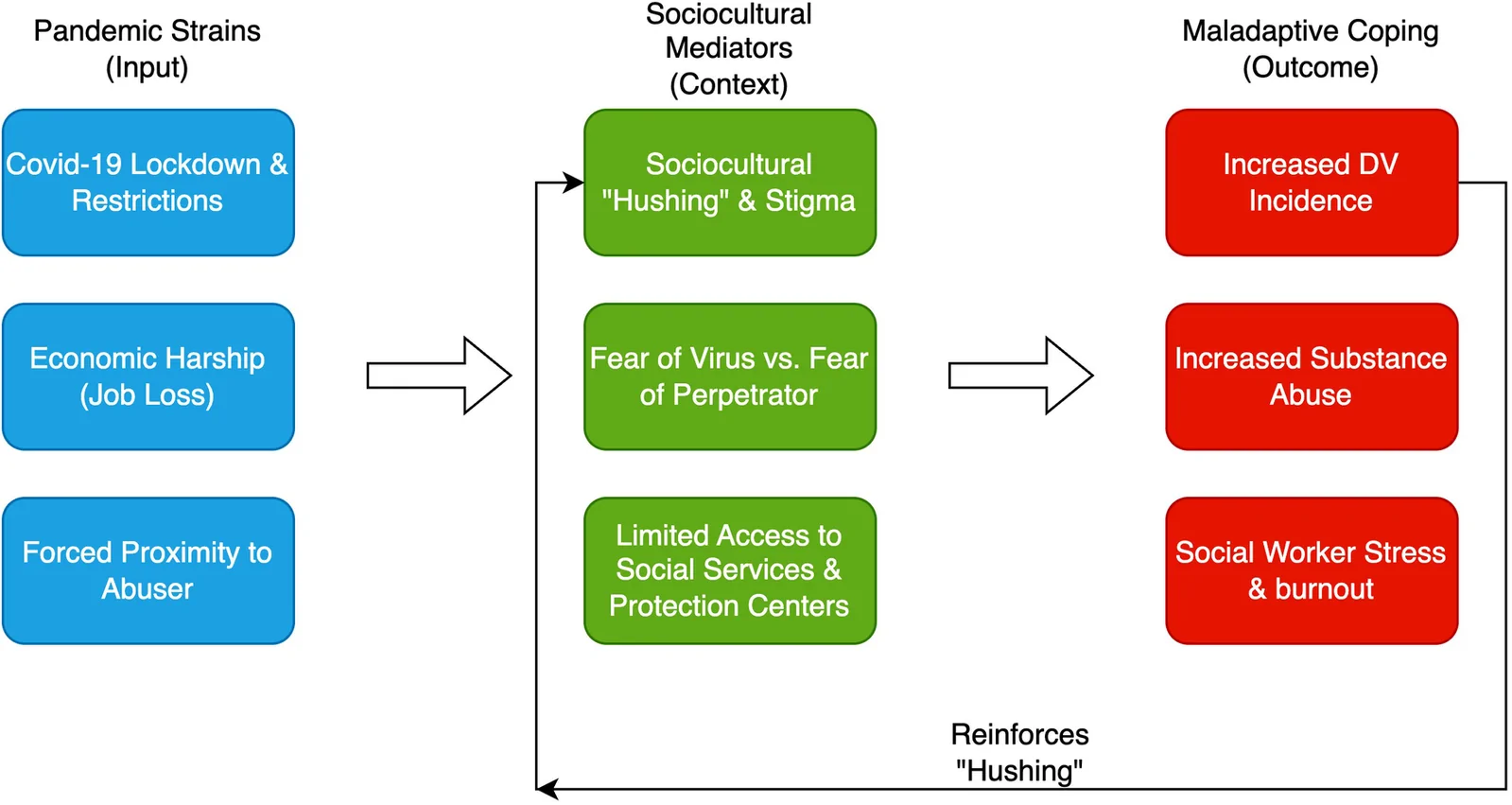
Conceptual framework of domestic violence (DV) in Jordan during COVID-19 pandemic: a social worker's perspective.

## Discussion

The latent content analysis of the interviews showed how COVID-19 has broken the social protection worker's ability to access and help victims in the centers covering Amman city. The biggest challenge for abused women was accessing FJPD Centers because of the lockdown and lack of transportation. Most of the abused women had contacted those centers through the phone or social media. The findings of this study showed that the group most exposed to violence were women between the age of 20 and 40. A recent study found that 78% of calls to police reporting DV come from the victim themselves, and in only 7% of those cases the suspect was on the scene when officers arrived (38). While only 8% of the calls reporting DV to law enforcement come from a neighbor or bystander who often do not face the same barriers to reporting to authorities that the victim experience (38). Family violence victim-serving agencies must explore new expanded community partnerships and encourage them to check on their neighbors, friends, and family (while maintaining adherence to any distancing regulations) and report any concerns they see or hear to the proper authorities (11, 38).

Additionally, our study showed that, there was an increase in the number but not the severity of cases of violence during the pandemic, this increase in demand could be a direct result of the increase in exposure to the abusers in isolation, this is consistent with the worldwide data reporting an increase in DV during the quarantine. The GST (39) could explain the increase of DV during the COVID-19 pandemic. Based on this theory, strain could initiate negative emotions, that might increase the likelihood of criminal behavior. There are three sources of strain: failure to achieve goals represented by economic loss, removal of positive stimuli represented by loss of social support and mobility, and the presentation of noxious stimuli represented by enforced proximity to an abuser (39, 40).

DV is a negative approach to coping with strain-generated negative feelings, such as anger, anxiety, and depression. Quarantine, restrictions of mobility and economic hardships could be the underlying causes of the negative feelings that led to the increase of DV during the pandemic.

Thus, interventions should go beyond immediate protection to address the underlying socio-economic strains that trigger DV.

Although the quarantine aimed to protect Jordanians health, still the socioeconomic implications of the COVID-19 restrictions measures inflicted the lives of individuals and households. The Jordanian government developed social programs to address the vulnerabilities and the negative consequences of the pandemic (41). However, this could not provide compensation to all for those who lost their jobs and their ability to provide for their families. According to Abuhammad, 2020; the only predictors of violence amongst women were unemployed and marital status (14). Many women during the COVID-19 outbreak lost their jobs and based on the literature; unemployed women are more at risk to experience violence than employed women. The stressful economic situation pushed some couples to abuse alcohol and drugs, which are known factors that increase the incidents of violence (42–44). DV causes economic losses at the individual, society and country level due to the provision of providing means to protect victims, treatment of the injured women, and the loss of some manpower that was actively contributing to the growth of the economy (45).

The phenomenon of social hushing, described by some social workers “she hides that to protect her family”, reflects a localized socio-cultural context where maintaining domestic privacy and family reputation is often prioritized over seeking formal intervention and individual safety.

Also, the victims fear of the virus more than the abuser turned government's safety measures into a tool that the abuser used to trap the victim. Lastly, the decrease in economic resources caused conflict between partners, which led to a threat to the traditional male provider identity and subsequently to a violent attempt to reclaim control in the home. Our findings go in line with a systematic review conducted on Jordanian articles, which categorized the multiple forms and socio-economic causes of DV in Jordan. The identified causes included economic, socio-cultural, and psychological factors associated with the pandemic and lockdowns, such as poverty, job loss, male dominance, reduced income, fear, and stress, among others (46). A study from Jordan established a prevalence of 40% DV in Jordan especially among married and unemployed women during the pandemic. Our study identified the associated causes and theories of such a high prevalence of DV (14).

While studies have identified silence and “social hushing” as forms of resistance used by women, our findings suggest that during the COVID-19 pandemic, this was no longer a choice but a systemic entrapment, that stripped women of the resistance strategies (47).

Studies on Swedish social workers' showed that in order for them to adapt to the new circumstances associated with the pandemic, they used “shared trauma” with clients and their previous knowledge and work experience. However, in Jordan, our study showed that entrapment and patriarchal power dynamics forced victims to choose between viral exposure and domestic abuse. This have left the exhausted social workers with few options to help the victims (48).

On the other hand, a study from Nepal found that violence against women is normalized through sociocultural traditions like dowry and child marriage. Women there usually have a fear of reporting due to potential retaliation and this leads to severe mental health issues. The authors suggested that effective intervention requires confidential, individualized counseling and shifting social norms to empower women to safely access support and help (49).

Our findings mirror the 2014 West Africa Ebola virus epidemic and natural disasters like Hurricane Katrina, where the focus on a public health emergency led to the neglect of women's safety, resulting in disproportionate harm to women and girls. These also demonstrated that the stress of property damage and resource loss significantly increases the likelihood of physical violence as a method of conflict resolution. Unlike the findings in Western disaster studies where reporting often remains a primary option, our study indicates that the pandemic weaponized existing social stigmas. This trend suggests that in conservative societies, public health crises do not just create new barriers, but they amplify existing cultural ones (50–53).

Our findings closely mirror regional and international evidence regarding the mechanics of DV during COVID-19 period. Similar to observations across Arab countries and Western settings, abusers in Jordan actively weaponized viral anxiety and mobility restrictions to control their victims. The reporting dynamics described by social workers, where strict curfews suppressed formal contact, are similar to what was reported by frontline service providers globally. Furthermore, the reliance on “hushing” and silence, and informal coping strategies aligns with established patterns of Jordanian women's resistance within patriarchal structures. This reflects the profound socio-cultural barriers, disrupted informal support networks, and help-seeking challenges (54–60).

The inclusion of both male (*n* = 11) and female (*n* = 9) social workers provided a balanced and deeper analytical view of the pandemic impact on DV, both on the victims and their abusers. While female social workers highlighted victims fears and entrapment, male workers focused on the strains affecting the perpetrators, such as job loss, poverty and consequent loss of the provider role.

This study represents one of the first qualitative investigations to explore the unique, dual-level systemic perspectives of social workers and center managers to better understand the DV problem during COVID-19 pandemic in Jordan. Purposively sampling male and female staff alongside managers provided a balanced, multi-level view of the pandemic's impact in addition to synthesizing broader systemic patterns. However, our findings cannot be generalized because of the qualitative design and due to some limitations, that should be taken into consideration when trying to understand this study. Social workers were chosen as key informants in this study because of their insider knowledge and access to data (61). Their specialized experience was indispensable to explore DV, which is a sensitive topic in the Jordanian context that victims and perpetrators cannot freely speak about. Direct victim engagement was not possible at that time due to strict confidentiality guidelines and ongoing safety risks to victims during the pandemic. Still, the fact that the interviews were conducted with social workers, not with the victims themselves might have influenced the description of the experiences of the victims. However, the researchers could not reach out to the victims because this is a taboo subject, and such data is confidential. Thus, the findings represent the professional interpretation of these experiences rather than first-hand point of view. Additionally, this study collected the data from the FJPD Centers in Amman governorate and further research might be needed to study the situation in other governorates. While Amman represents more than half of Jordan's population and encompasses diverse socio-economic strata, these findings may not represent the unique social norms, tighter family structures, or greater service accessibility barriers experienced by women in rural governorates. As this was cross-sectional retrospective study between February and July 2022 following the lifting of restrictions in late 2021, participant contributions may be subject to recall bias and the findings represent only the crisis period, preventing the evaluation of evolving post-pandemic dynamics. Lastly, the research team consisted of bilingual Jordanian academics familiar with social, cultural and economic background of Amman – Jordan, who analyzed Arabic qualitative transcripts using English coding. This allowed for deeper understanding of local phrases and practices. However, this introduced cross-language translation in addition to interpretation bias by confirming preexisting beliefs. In order to address these biases, the researchers applied back-translation and practiced reflexivity by discussing and challenging one another's interpretations of the data (62).

Several challenges were faced during this study. The researchers encountered significant administrative obstacles in obtaining institutional permissions to access the protection centers, which reflects the sensitive nature of this subject. Moreover, strict security and confidentiality protocols limited audio recording to only four interviews, requiring intensive manual notetaking for the remainder. Despite using a dual-researcher note-taking and verification protocol, relying on handwritten notes may have missed exact phrasing and subtle nuances compared to audio recordings.

## Conclusion

Viewed retrospectively, this research tried to take a look inside social workers' minds to help us gain a deeper understanding of the violence against women during COVID-19 crisis. Upon exploring the situation from their perspectives, the findings suggest that the pandemic did not create new behaviors but it exacerbated existing vulnerabilities and its psychological and financial effects. Understanding this dynamic is essential to ensure that these factors do not remain a barrier in future socio-economic or public health crises. Those results should be taken into consideration because they can help to decrease or even prevent victims from being abused by providing a fast and feasible way to access FJPD services and providing economical support to decrease the incidence of drug abuse and its implications. Further studies, especially with the abusers and victims could help us come up with better and more effective solutions to this issue.

## Recommendations

DV with its devastating consequences on victims' lives must be combated by facilitating and developing effective reporting methods, creating protection interventions including safe shelters for the victims, developing schooling systems that teach children the importance of being kind and respectful towards women. Additionally, women should be taught about the devastating consequences of DV and learn how to protect themselves. Women should be raised to believe in their self-value and not be afraid of reporting DV. The stress, fear, economic and psychological pressure that happen to people during crisis and lockdowns make fertile soil for exacerbating DV. Actions such as social distancing, restricted movement and closures of key community resources are likely to dramatically increase the risk of family violence. Government, local societies, non-governmental organization in cooperation with the media are advised to spread awareness about the increase in cases of DV and the impacts of that on individuals and society. In addition, they should provide physical, economical, and psychosocial help that the victims might need. Social workers have a valuable role in providing first-line psychological counseling, including listening empathetically without judgment, enquiring about the needs and concerns, validating peoples' experiences and feelings, enhancing safety, and connecting people to relevant support services.

To overcome the possible physical and socio-economic barriers of reporting, the FJPD should utilize technology in digital reporting tools, improve access to social services and develop community-based safe shelters that do not rely on centralized transportation. This could be done by using silent reporting applications with disguised interfaces that allow the victims to request help without alerting the abuser and free of charge access to emergency web portals ensuring that the socio-economic hardships do not prevent them from seeking help. Moreover, social workers should be trained on standardized tele-counseling protocols via encrypted platforms. This will make sure that the process remains safe, private, and accessible by vulnerable victims.

While digital solutions are valuable, they are structurally unfeasible as standalone tools for less privileged women, who often lack private smartphones, internet access, or digital literacy. Thus, digital reporting must be paired with low-tech, zero-tariff phone messaging options that do not relay on internet to ensure feasibility within the Jordanian context. Furthermore, reaching women completely excluded from technology requires integrating decentralized digital access points into trusted physical spaces like local pharmacies, clinics, and community centers.

Lastly, future studies should focus on the experiences of the victims, to better understand their perspective, conduct longitudinal studies to track the long-term psychological impact of DV and compare between rural and urban areas to assess for geographic disparities and socio-cultural differences impact DV reporting.

## Data Availability

The raw data supporting the conclusions of this article will be made available by the authors, without undue reservation.
