# Supplementary material for: “Nowhere to go” qualitative exploration of social workers' perspectives on violence against women in Jordan during the COVID-19 pandemic
Source: Front Glob Womens Health. 2026 Sep 24;7:1899338. doi: 10.3389/fgwh.2026.1899338 (PMC13646062; doi:10.3389/fgwh.2026.1899338)
Supplement: Supplementary file 1 [file Datasheet1.pdf]

Appendix

Social workers' perceptions of domestic violence against women during the Covid-19 pandemic in Jordan: A qualitative study

Researcher: Hana Taha

This study aims to understand the impact of the coronavirus crisis on domestic violence against women in Jordan during the Covid-19 pandemic in 2020.
The United Nations defines domestic violence against women as "a pattern of behavior in any relationship that is used to gain or maintain power and control over an intimate partner. Abuse is physical, sexual, emotional, economic or psychological actions or threats of actions that influence another person. This includes any behaviors that frighten, intimidate, terrorize, manipulate, hurt, humiliate, blame, injure, or wound someone. Domestic abuse can happen to anyone of any race, age, sexual orientation, religion, or gender. It can occur within a range of relationships including couples who are married or engaged. Domestic violence affects people of all socioeconomic backgrounds and education levels".

We would appreciate having 30 minutes of your time to learn more about your experiences and thoughts related to the research topic. Please read the answers to the below questions before deciding whether to participate in this study.

- **Can I withdraw later if I want to?**
  Yes. Participation is entirely voluntary. If you decide to participate, you will be asked to sign a consent form. However, you remain free to change your mind and withdraw at any time without giving a reason. Your decision not to participate or to withdraw will be respected and will not affect you in any way.
- **Will my participation be confidential?**
  Yes. Your participation will be completely confidential. All personal information will remain anonymous and securely stored. Any data shared within the research team will be coded numerically (instead of names or addresses) so that no participant can be identified.
- **Are there any disadvantages or risks to participating?**
  No. There are no disadvantages or risks associated with participating in this study, either short-term or long-term.
- **What will you do with the information collected?**
  The data and texts will be translated into English and analyzed by the research team to write a scientific article aimed at combating violence against women.
- **Do you have approval to conduct this study in Jordan?**
  Yes. The study has been reviewed and received ethical approval from the Research Ethics Committee at Hashemite University.

If you wish to participate in this study, please sign this consent form. Thank you for your cooperation.

Signature:

Participant data:

Age:

Sex:

Job:

Education:

Interview guide:

1. Starting from March 2020 and due to the Covid -19 pandemics restrictions measures in Jordan, what are the challenges that abused women encountered trying to reach you during the lockdown?
2. Who were the most vulnerable women to domestic violence?
3. How did the victims reach your organization during the pandemic?
4. Can you describe the nature and extent of violence against the women before and during the pandemic?
5. What are the potential causes or risk factors that lead to the increase of reported cases?
6. Can you describe the socioeconomic status of the abused women?
7. Did the victims or their partners used drugs or alcohol? if yes, did their consumption increase during the pandemic and how did it affect the victims?
8. How did you deal and followed up the cases of the women who reached out to you?
9. What were the barriers of reporting and access to care? How did you handle those obstacles?
10. What are your suggestions for the prevention of violence against women during crisis?
